# DETERMINING EVIDENCE FOR EFFECTIVE FAMILY CAREGIVER COMMUNICATION: STRATEGIES LEADING TO BREAKDOWN AND REPAIR

**DOI:** 10.1093/geroni/igac059.1151

**Published:** 2022-12-20

**Authors:** Carissa Coleman, Jinxiang Hu, Kristine Williams

**Affiliations:** University of Kansas, Kansas City, Kansas, United States; University of Kansas Medical Center, Kansas City, Kansas, United States; University of Kansas Medical Center, Lawrence, Kansas, United States

## Abstract

Communication is fundamental for dementia care and identifying evidence for strategies that facilitate or impede communication is needed. We analyzed 221 videos from a randomized controlled trial of a family caregiver telehealth intervention (FamTechCare) using Noldus second-by-second behavioral coding of communication behaviors and breakdowns in interactions between 53 caregiver-person with dementia dyads. Coded data from 3,642 30-second observations were first analyzed using penalized regression for feature selection (least absolute shrinkage and selection operator; LASSO) to identify strategies most important for predicting prevention and successful repair of communication breakdown. Bayesian mixed modeling was then used to identify communication strategies associated with successful versus unsuccessful prevention and repair of communication breakdown taking into account of the dyadic structure of our data. Results showed that given our data, communication breakdown was associated with caregivers changing topic (Mdn=11.23, 95% CrI [4.39, 24.43]), ignoring (Mdn=11.47, 95% CrI [4.75, 24.17]), making commands (Mdn=10.55, 95% CrI [3.41, 23.22]) and taking over the task (Mdn=4.04, 95% CrI [1.69, 7.23]). Successful repair of breakdown was associated with caregivers verbalizing understanding (Mdn=-0.47, 95% CrI [-0.88, -0.09]), tag questions, (Mdn=-2.44, 95% CrI [-5.35, -0.33), and silence (Mdn=-0.78, 95% Crl [-1.18, -0.40) while ignoring and changing topic were associated with unsuccessful repair (Mdn=3.63, 95% Crl [-2.56, 4.78; and 2.51 [1.39, 3.74]). These results provide evidence for development and testing of evidence-based communication strategy training for family caregivers of persons with dementia. Future analyses will identify effects of dementia stage, diagnosis, and dyad characteristics on associations.

